# A Novel Immunodominant CD8+ T Cell Response Restricted by a Common HLA-C Allele Targets a Conserved Region of Gag HIV-1 Clade CRF01_AE Infected Thais

**DOI:** 10.1371/journal.pone.0023603

**Published:** 2011-08-22

**Authors:** Supranee Buranapraditkun, Ursula Hempel, Patrawadee Pitakpolrat, Rachel L. Allgaier, Pattarawat Thantivorasit, Sven-Iver Lorenzen, Sunee Sirivichayakul, William H. Hildebrand, Marcus Altfeld, Christian Brander, Bruce D. Walker, Praphan Phanuphak, Pokrath Hansasuta, Sarah L. Rowland-Jones, Todd M. Allen, Kiat Ruxrungtham

**Affiliations:** 1 Vaccine and Cellular Immunology Laboratory, Chulalongkorn Medical Research Center (ChulaMRC), and Department of Medicine, Faculty of Medicine, Chulalongkorn University, Bangkok, Thailand; 2 Ragon Institute of MGH, MIT and Harvard, Boston, Massachusetts, United States of America; 3 Department of Microbiology and Immunology, Health Sciences Center, University of Oklahoma, Oklahoma City, Oklahoma, United States of America; 4 AIDS Research Institute IrsiCaixa - HIVACAT, Hospital Germans Trias i Pujol, Badalona, and Institució Catalana de Recerca i Estudis Avançats (ICREA), Barcelona, Spain; 5 Thai Red Cross AIDS Research Center, Bangkok, Thailand; 6 Department of Microbiology, Faculty of Medicine, Chulalongkorn University, Bangkok, Thailand; 7 Medical Research Council, Human Immunology Unit, Weather all Institute of Molecular Medicine, John Radcliffe Hospital, Oxford, United Kingdom; 8 Interdisciplinary Program of Medical Microbiology, Graduate School, Chulalongkorn University, Bangkok, Thailand; Karolinska Institutet, Sweden

## Abstract

**Background:**

CD8+ T cell responses play an important role in the control of HIV-1. The extensive sequence diversity of HIV-1 represents a critical hurdle to developing an effective HIV-1 vaccine, and it is likely that regional-specific vaccine strains will be required to overcome the diversity of the different HIV-1 clades distributed world-wide. Unfortunately, little is known about the CD8+ T cell responses against CRF01_AE, which is responsible for the majority of infections in Southeast Asia.

**Methodology/Principal Findings:**

To identify dominant CD8+ T cell responses recognized in HIV-1 clade CRF01_AE infected subjects we drew upon data from an immunological screen of 100 HIV-1 clade CRF01_AE infected subjects using IFN-gamma ELISpot to characterize a novel immunodominant CD8+ T cell response in HIV-1 Gag restricted by HLA-Cw*0102 (p24, _277_YSPVSILDI_285_, YI9). Over 75% of Cw*0102+ve subjects targeted this epitope, representing the strongest response in more than a third of these individuals. This novel CD8 epitope was located in a highly conserved region of HIV-1 Gag known to contain immunodominant CD8 epitopes, which are restricted by HLA-B*57 and -B*27 in clade B infection. Nonetheless, viral escape in this epitope was frequently observed in Cw*0102+ve subjects, suggestive of strong selection pressure being exerted by this common CD8+ T cell response.

**Conclusions/Significance:**

As HLA-Cw*0102 is frequently expressed in the Thai population (allelic frequency of 16.8%), this immunodominant Cw*0102-restricted Gag epitope may represent an attractive candidate for vaccines specific to CRF01_AE and may help facilitate further studies of immunopathogenesis in this understudied HIV-1 clade.

## Introduction

Treatment of HIV-1 infections with antiretroviral therapy has had an enormous impact on delaying disease progression in HIV-1 infections [1]. Unfortunately, the cost and delivery of these live-saving drugs remain substantial hurdles to providing access to all infected individuals, especially those in developing countries which account for a majority of all cases [2]. Furthermore, drug resistance and side effects continue to be problematic even though new antiretroviral treatment agents are being perpetually developed [3]. Therefore, the design of an effective HIV-1 vaccine remains a priority.

It is believed that the cellular immune response [4]–[7], in addition to neutralizing antibodies, plays a key role in the control of HIV [8]–[10]. Their ability to recognize infected cells depends upon the presentation of viral peptides by host human leukocyte antigen (HLA) molecules on the surface of the cell. Each HLA molecule binds a very specific set of 8–12 amino acid peptides, or epitopes, derived from intracellular viral proteins. The HLA molecules represent a highly diverse set of proteins, which can vary considerably between different ethnic populations [11], [12].

As a result of the specificity of HLA binding of viral peptides, HIV-1 is capable of evading many CD8+ T cell responses through single amino acid mutations in the epitopes [13], [14]. It is known that while some regions of the virus are highly diverse [15], other regions remain highly conserved due to preservation of critical functional or structural domains [16]–[19]. Therefore, there has been much focus on the identification of strongly and frequently targeted CD8 epitopes from highly conserved regions of the virus that would be refractory to viral escape due to high fitness costs. Although substantial CD8 epitopes restricted by HLA-A and -B alleles have been identified, to date very few HLA-C-restricted epitopes have been reported (http://www.hiv.lanl.gov/content/immunology). As such, the potential of HLA-C restricted responses may be underappreciated especially given the ability of HIV-1 Nef to selectively down-regulate HLA-A and -B restricted CTL responses [9]. As well, single nucleotide polymorphisms near the HLA-C gene locus have recently shown to be associated with a lower HIV viral set point [20]. More specifically, individuals with a variant 35 kb upstream of the *HLA-C* loci (*-35C/T*) which results in higher –expression of *HLA-C* alleles, exhibit enhanced control of viremia as compared to those with low *HLA-C* expressing alleles [21]. HLA-C polymorphisms may also have a direct effect on HIV infectivity and replicative capacity [22]. As such, further exploration of the role of HLA-C alleles in HIV infection is warranted.

A major hurdle in the design of an effective HIV-1 vaccine is the vast inter-clade sequence diversity of HIV-1, compounded by the numerous distinct clades distributed globally. To contend with this diversity, it is believed that clade-specific vaccines will be needed [15]. In Thailand, **t**he predominant strain of HIV-1 is the circulating recombinant form CRF01_AE, which accounts for between 90 and 95% of infections [23]. According to UNAIDS, HIV prevalence in Thailand is estimated at about 1.4% in adults of 15 to 49 years (0.7–2.1%) (http://www.unaids.org). In addition to Thailand, CRF01_AE is also prominent in other countries of Southeast Asia (except India), where it accounts for approximately 84% of all infections [24]. Some studies suggest that HIV-1 clade CRF01_AE infected individuals show faster progression rates to AIDS and shorter median survival than individuals from cohorts located in Eastern and Southern Africa or high-income countries, but similar to those with clade D infection in Sub-Saharan Africa [25], [26].

To date little is known about the cellular immune response against CRF01_AE [27]–[29]. This impairs the identification of promising epitope dense regions of the virus for inclusion into vaccines and the study of its pathogenesis. In this report, we have identified a frequently targeted novel immunodominant CD8 epitope in Gag in HIV-1 clade CRF01_AE infected subjects from Thailand. This novel epitope is restricted by HLA-Cw*0102, a frequent HLA-C allele in the Thai population (allelic frequency of 16.8%, http://www.ncbi.nlm.nih.gov). Even though the specified epitope lies in a highly conserved region of Gag, HIV-1 was found to frequently escape from this CTL response suggestive of a strong selection pressure. The identification of frequently targeted epitopes, especially in conserved regions, will be important in the design of HIV vaccines.

## Results

### Identification of a commonly targeted CD8 epitope in Gag in CRF01_AE infected subjects

To identify commonly targeted CD8+ T cell epitopes in the Thai population, we drew upon data from an immunological screen of a cohort of 100 treatment naïve, HIV-1 clade CRF01_AE infected subjects (Table 1) using an IFN-γ ELISpot assay and overlapping 18-mer peptides spanning the entire HIV-1 clade CRF01_AE genome (Buranapraditkun et al, manuscript in preparation). Infection with CRF01_AE was confirmed through Gag sequencing. One overlapping peptide (OLP-038; _273_IVRMYSPVSILDIRQGPK_290_) in Gag was recognized by 29 subjects (the 7th most frequently targeted OLP), with an average of 1,428.3 SFCs in responders. In 9 (31%) subjects with a detectable response against OLP-038 this also represented the overall strongest response.

**Table 1:** Clinical characteristics of HIV-1 clade CRF01_AE infected subjects (N = 100).

	Total	Cw*0102+ve	Cw*0102-ve
Number of patients	100	33	67
Female	47	15 (45%)	32 (48%)
Male	53	18 (55%)	35 (52%)
Median age	32	31	32
Median sero-diagnosis (year range)	1.4 (0–14.8)	1.4 (0–7.4)	1.4 (0–14.8)
Median CD4+ T cells (cells/mm^3^)	272	227	284
Median plasma HIV-1 RNA (copies/ml)	23,430	29,778	22,029

This epitope is located within an epitope-dense region of p24 which includes epitopes restricted by various HLA alleles including -A*02, -B*52, and -Cw*18 (http://www.hiv.lanl.gov/content/immunology). None of these alleles were predominantly expressed in responders. However, 25 of the 29 subjects (86.2%) mounting detectable IFN-γ T cell responses against OLP-038 expressed HLA-Cw*0102 (p = 8.74*10^−13^).

**Figure 1:**
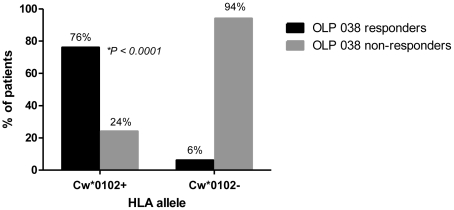
Predominant recognition of OLP-038 in Cw*0102+ve subjects. ELISpot responses in Cw0102+ve and Cw0102-ve subjects were tested. The majority of Cw*0102+ve subjects (25/33; 76 percent) exhibited an ELISpot response against OLP-038, versus a minority of the Cw*0102-ve subjects (4/67; 6 percent); *p*<0.0001.

Conversely, of the 33 Cw*0102+ve subjects in our cohort, 25 (75.8%) had a detectable response against OLP-038, ranging from 230 to 3,990 SFCs (the mean of SFC in the responders was 1,561) (Fig. 1) making it the most frequently targeted peptide in the Cw*0102+ve subjects, vs. 6% recognition (4 subjects) in the Cw*0102-ve subjects with an average of 598 SFCs.

### Definition of the novel Cw*0102-restricted CD8 epitope p24 _277_YI9_285_

**Figure 2:**
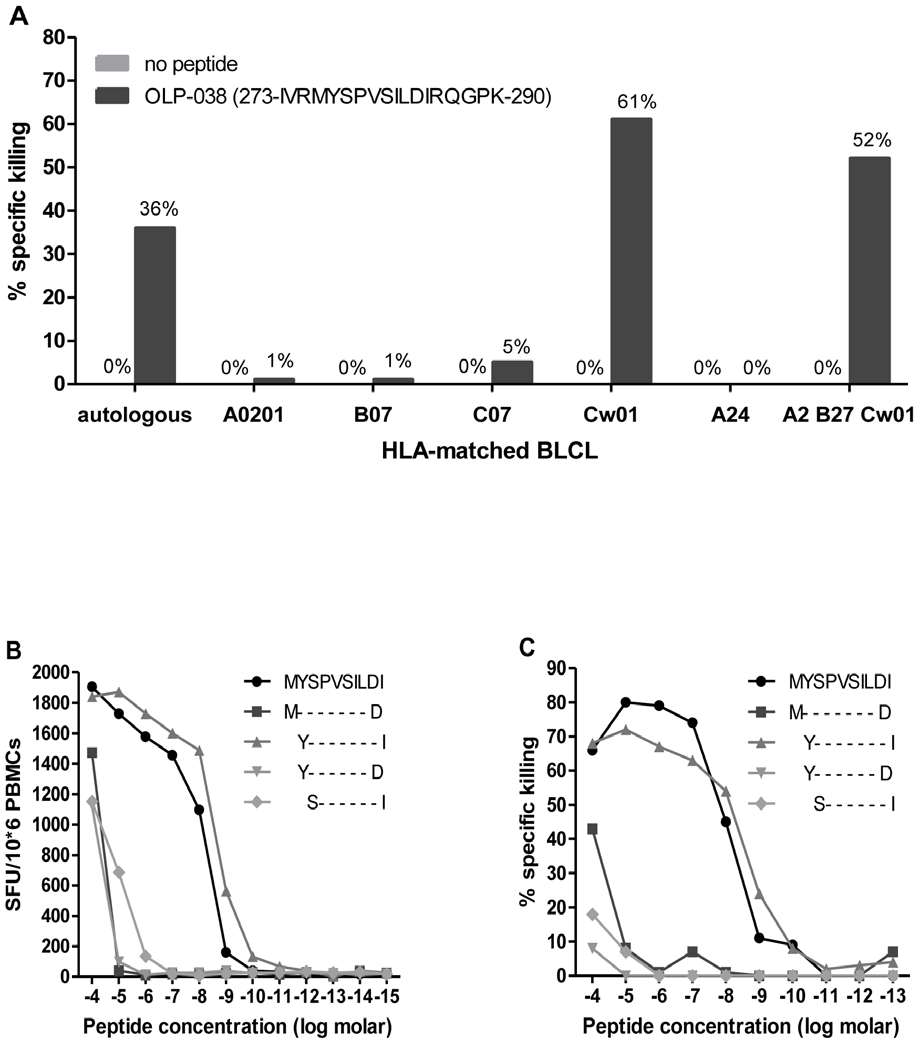
Identification of the novel Cw*0102-restricted CD8 epitope _277_YI9_285_. (A) Autologous BLCL from subject 0578 who strongly responded to OLP-038, and BLCL targets from 6 other individuals expressing one of subject 0578 HLA alleles, were tested for their ability to recognize peptide-pulsed targets using a ^51^Cr-release assay. Responses were only detected in the presence of HLA-Cw*0102, confirming this as the restricting HLA allele. The optimal CD8 epitope within OLP-038 was then fined mapped using both (B) ELISpot and (C) ^51^Cr-release assays, identifying the 9-mer YI9 as the minimal optimal epitope.

To define the restricting HLA allele, we conducted ELISpot and Chromium-release assays. In the ^51^Cr-release assay, PBMC from subject 0578 recognized OLP-038 only when presented by BLCL from subjects expressing HLA-Cw*0102 (Fig. 2A). We then conducted fine mapping of the epitope using serial dilutions of peptide truncations in both IFN-γ ELISpot (Fig. 2B) and ^51^Cr-release assay (Fig. 2C). These assays established the minimal optimal epitope as the 9-mer _277_YSPVSILDI_285_ or YI9, although notably the longer peptide _276_MYSPVSILDI_285_ was nearly equally reactive. CD8-restriction of this epitope was also confirmed using CD4 and CD8 depletions (data not shown).

### Frequent viral escape from the OLP-38 epitope in Cw*0102+ve subjects

**Figure 3:**
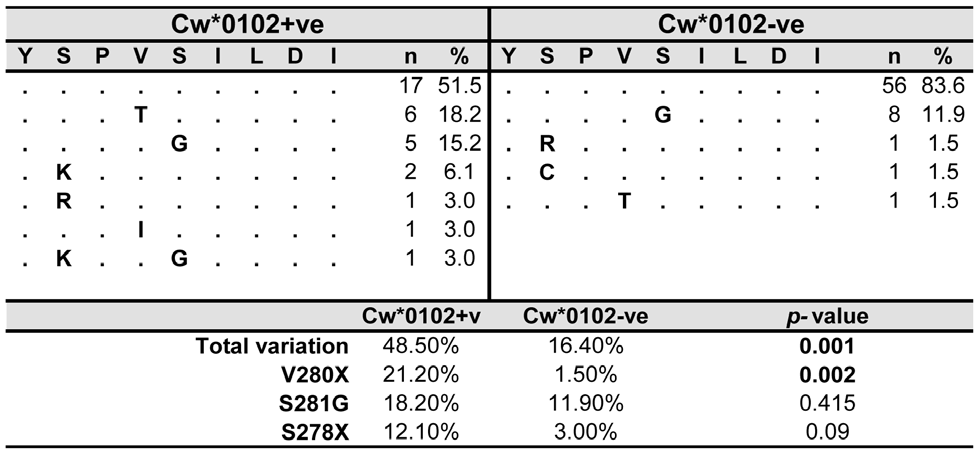
Cw*0102 associated footprints in the YI9 epitope. Gag sequences were generated from all 100 subjects and sequence variation within the YI9 epitope compared between Cw*0102+ve and Cw*0102-ve subjects. Mutations in YI9 were significantly elevated in subjects expressing Cw*0102 (p = 0.001), with the V280X and S281G mutations predominating.

To determine whether YI9-restricted CD8 responses were capable of exerting immune selection pressure upon HIV-1, sequence data of the Gag-region was generated from all subjects. As shown in Fig. 3, irrespective of the expression of Cw*0102, 73/100 (73%) sequences from this cohort were conserved. This is nearly identical to an analysis of 437 CRF01_AE sequences from Thailand derived from the LANL HIV Sequence Database, in which 72% of sequences were conserved. Notably, in the absence of Cw*0102, the number of consensus sequences increased to 56/67 (83%), while in contrast we observed a strong association between mutations in YI9 and the expression of Cw*0102. More specifically, in Cw*0102+ subjects 16/33 (48%) exhibited mutations in YI9 versus only 11/67 (16%) in Cw*0102-ve subjects (p = 0.0015). In Cw*0102 subjects with a wildtype sequence at YI9 we detected a response rate of 100% against OLP-038, with an average ELISpot response of 1,832 SFCs. In contrast, only 50% of Cw*0102 subjects exhibiting mutations in YI9 mounted detectable responses against this epitope, and with a lower average magnitude of 986 SFCs in responders (p<0.001). While mutations occurred at positions P2, P4 and P5, the most frequent mutation was a single amino acid substitution at position 4, mutation V280X (predominantly Threonine). Mutations at P4 reduced the response rate to 71.4% and diminished the magnitude of the ELISpot responses to an average of 682 SFCs in responders (p<0.001). The amino acid mutation at P5, S281G, reduced the ELISpot response rate to 50%, with an average magnitude of the ELISpot response of 1,493 SFCs in responders (p<0.001). More dramatically, mutations at P2, which occurred in four cases, appeared to completely impair ELISpot responses (p<0.001). No significant difference in VL or CD4 counts could be found however between the different groups.

**Figure 4:**
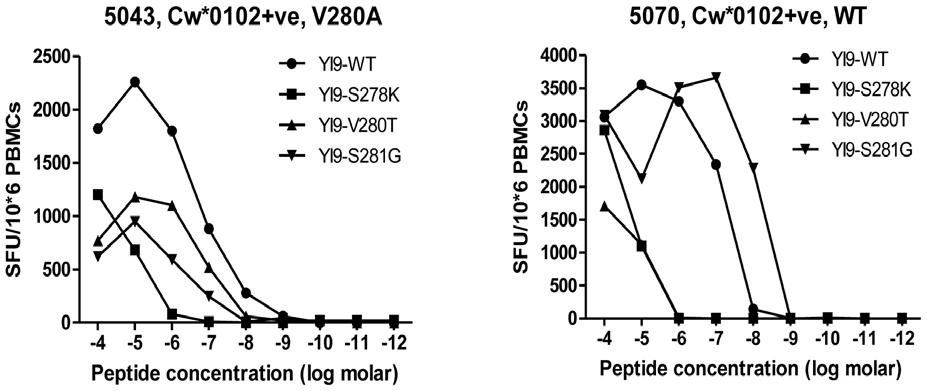
Cw*0102-associated mutations in YI9 impair CD8+ T cell recognition. BLCL from Cw*0102+ve subjects 5043 and 5070 were pulsed with serial dilutions of wildtype and variant forms of YI9 and tested for recognition by ELISpot assay. P2 mutation S278K, P4 mutation V280T, and P5 mutation S281G all impaired recognition, with the P2 anchor mutation S278K exhibiting consistently the most significant impairment.

Finally, ELISpot assays using mutated peptides were conducted on PBMC from two additional Cw*0102-positive patients not originally included in the primary cohort due to a lack of sequencing data. In both patients we were able to illustrate a loss of recognition for both of the peptides with the V280T and S278K mutation (Fig. 4), illustrating the direct effect of these Cw*0102-associated mutations on CD8+ T cell recognition. Subsequent sequencing of these subjects showed a V280A mutation in subject 5043 (not tested), and a wildtype sequence in subject 5070. In summary, these data define YI9 as an immunodominant and novel Gag epitope in the Thai population restricted by the common HLA-Cw*0102 allele.

## Discussion

This study identifies a previously unknown HLA-Cw*0102 restricted CD8 epitope _277_YSPVSILDI_285_ (YI9) located in p24 Gag. Although the overlapping peptide OLP-38 containing YI9 was only the 7^th^ most frequently targeted epitope in this cohort (Buranapraditkun et al, manuscript in preparation), it exhibited the clearest HLA restriction of all dominantly targeted OLPs. Indeed, over 86% of subjects with detectable ELISpot responses against OLP-38 expressed Cw*0102.

Despite the location of the YI9 epitope in a highly conserved region of p24 Gag, mutations in YI9 were frequently observed in this epitope in nearly 50% of all Cw*0102+ve subjects. The most frequently occurring mutation was a V280T mutation at position 4 (18% of all Cw*0102+ve subjects), which was all but absent in Cw*0102-ve subjects. The frequency of Cw*0102 in the Thai population, and the frequent escape observed in this epitope, suggests the likely reversion of this escape mutation back to wildtype upon transmission to a new host. These data may suggest the ability of this mutation to impair viral replication as observed for other escape mutations in this region of p24 [30], [31]. Position 2 represents an anchor position for binding of the peptide to the HLA class I groove, and thus a potentially more promising pathway to viral escape. Here, mutations at position 2 were present in 12% of all Cw*0102+ve subjects and again rarely in Cw*0102-ve subjects.

The frequent recognition of the YI9 epitope (76%) observed in this cohort suggests it to be amongst the most immunodominant in CRF01_AE infections. In comparison to extensive immunodominance data available for clade B infection [32], only a handful of CD8 epitopes are as commonly targeted as YI9 in acute or chronic infection [33]. Notably, this peptide maps close to the region of Gag containing immunodominant CD8 epitopes restricted by HLA-B*27 (_263_KRWIILGLNK_272_) and HLA-B*57 (_240_TSTLQEQIGW_249_). Both of these HLA alleles are associated with a beneficial outcome in clade B infections [17], [32], [34]–[36]. However, in our cohort there is no significant difference in viral loads between Cw*0102+ve and Cw*0102-ve patients (mean 71,184.5 vs. 69,732.2 copies/ml, p = 0.89), nor in CD4 T cell counts (mean 243.6 vs. 292.6 cells/mm^3^, p = 0.278). This may be the result of the high rate of viral escape within this epitope [37].

Further analysis of the HLA data showed linkage disequilibrium between Cw*0102 with B*4601 (p = 8.55*10^−13^), another common HLA epitope in the Thai population (allelic frequency 17.2%, NCBI). While we saw significantly more mutations in YI9 in Cw*0102+ve subjects than in Cw*0102-ve subjects (p = 0.0015), there was no significant difference in the occurrence of YI9 mutations in B4601+ve versus B4601-ve patients (p = 0.197). Thus, this argues against B4601 as the restricting HLA type, as does the strong data showing restriction of this response in Cw*0102 positive cells (Fig. 2A).

HIV is known to down-regulate HLA-A and HLA-B alleles on the cell surface [38], leaving HLA-C intact in order to protect cells from attack by NK cells [39]. As such, HLA-A or HLA-B restricted CD8+ T cells may be partially compromised in their ability to recognize and destroy HIV-infected cells as compared to HLA-C restricted responses. However, few HLA-C restricted epitopes have been identified to date (http://www.hiv.lanl.gov/content/immunology/tables/ctl_summary.html). As such, a greater focus on the identification and characterization of HLA-C restricted responses may be warranted to aid in the development of an effective HIV vaccine.

Inactivated virus and protein-based vaccines have not been able to induce sufficient antibody responses that lead to immune control in HIV-1 infection [40]–[42]. Although to date all attempts to induce CD8+ T cell responses against HIV-1 that reduce viral load set point or even prevent infection have failed [43], recent results have demonstrated the importance of those responses for controlling HIV-1 responses [44]. Thus, it is important to identify immunodominant epitopes in HIV-1 that are commonly targeted. Given that an effective HIV vaccine will likely have to be clade specific [15], there are very few data on CD8 epitopes in clade CRF01_AE which is the dominant clade in Southeast Asia [45]. Therefore, increased knowledge of immunodominant CD8 epitopes and their correlation with viral control in CRF01_AE infection is warranted to shed more light upon T-cell based HIV vaccine developments in this region.

## Materials and Methods

### Ethics Statement

This study was approved by the Faculty of Medicine, Chulalongkorn University's Institutional Review Board (IRB) and the IRB at the Massachusetts General Hospital and all study subjects provided written informed consent.

### Subjects

100 HIV-1 clade CRF01_AE (as obtained by Gag sequence) infected Thai volunteers were enrolled at The Immune Clinic, The King Chulalongkorn Memorial Hospital and Anonymous Clinic, Thai Red Cross AIDS Research Centre in Bangkok, Thailand. Viral load, HLA type, Gag sequences and ELISpot data were collected from each of the individuals. All subjects were antiretroviral treatment naïve, with a suspected seroconversion between 1992 and 2007. CD4+ T cell counts ranged from 1 to 906 cells/mm^3^ (mean 294 cells/mm^3^), plasma HIV-RNA levels ranged from 162 to 1,040,830 copies/ml (mean 74,693 copies/ml), as determined by the Chiron b-DNA Monitor test (Quantiplex v3.0, Bayer). Plasma HIV-RNA levels greater than 500,000 copies/ml were tested by the Abbott Real Time PCR HIV-1 Assay (range 40–10,000,000). Clinical data are summarized in Table 1.

### HLA Typing

High resolution HLA Class I typing was performed by DNA Sequenced Based Typing (SBT) at The University of Oklahoma Health Sciences Center CLIA/ASHI certified high resolution HLA tying laboratory. Briefly, exons 2 and 3 of class I gene were amplified and DNA sequenced. Ambiguous types were resolved to 4 digits with either group specific amplification followed by DNA sequencing of the specifically amplified allele and/or by PEL-FREEZ/Invitrogen UNITRAY SSP kits.

### PBMC Isolation and BLCL preparation

Peripheral blood mononuclear cells (PBMC) were isolated by density gradient separation with Isoprep (Robbins Scientific Corporation, Sunnyvate, CA) and cryopreserved for further immunological assays. Briefly, after removal of plasma, ACD-treated whole blood was diluted 1∶1 with RPMI1640 medium containing 2 mM L-Glutamine (Gibco, USA) and layered over Isoprep. Samples were then centrifuged at 1,500 rpm for 30 min and the PBMC layer harvested and washed twice with RPMI1640. Harvested PBMC were then resuspensed with R10 medium (RPMI1640 supplemented with 100 U/ml of penicillin, 100 U/ml of streptomycin and 10% heat-inactivated fetal bovine serum [FBS, Bio Whittaker, Maryland, USA]). Epstein-Barr virus (EBV)-transformed B-lymphoblastoid cell lines (BLCL) were established from PBMC of each subject by incubation with supernatant of EBV for 1 hour at 37°C in 5%CO_2_. BLCL were maintained in R20 medium with 1 µg/ml cyclosporin A. The B95-8 cell line was kindly provided by The National Institute of Health (NIH) Thailand.

### Peptides

Based on sequences of HIV-1 CRF01_AE, available at the HIV immunology database (http://www.hiv.lanl.gov), a set of 413 overlapping peptides (OLPs) was generated. The peptides were 18-mers, overlapping by 10 amino acids and spanning the entire coding region of CRF01_AE. They were synthesized on an automated peptide synthesizer at the peptide synthesis facility of the Massachusetts General Hospital using 9-fluorenylmethyloxycarbonyl chemistry or were obtained from Research Genetics, Huntsville, Ala. The novel epitope, peptide YSPVSILDI (YI9), restricted by HLA-Cw*0102, was synthesized by f-moc chemistry and provided by Prof. Sarah Rowland-Jones, Weatherall Institute of Molecular Medicine, John Radcliffe Hospital, Oxford, UK.

### Generation of CTL lines by *in vitro* stimulation

10×10^6^ PBMC were cultured with peptide at 10 µg/ml for 1 h, washed, and placed in 24-well plates at 5×10^6^ cells/ml in R10 containing 330 U/ml of IL-7 (Genzyme). On days 3, 7 and 10, 100 U/ml of IL-2 (kindly provided by NIH, USA) was added to cultures and CTL activity assessed on day 14.

### ELISpot assays

96-well ELISpot plates (MAIP S45; Millipore, Bedford, Mass.) were coated with 100 µl/well of 2 µg/ml of mouse anti-human interferon-gamma (IFN-γ) monoclonal antibody (mAb) 1-D1k (Mabtech, Stockholm, Sweden) in PBS and incubated at 37°C in 5% CO_2_ for 3 h. The plates were then washed six times with 200 µl PBS/well and blocked with 200 µl R10/well for 1 h at room temperature. PBMCs were added to the plates at 100,000 cells/well and peptides added at a final concentration of 2 µg/ml. Culture medium alone served as a negative and PHA-P at final concentration of 10 µg/ml as a positive control. After incubation for 16 h at 37°C in 5% CO_2_, the plates were washed manually 6 times with 200 µl/well of PBS/0.05% Tween and once with 200 µl/well of PBS. 50 µl/well of 1 µg/ml mouse anti-human IFN-γ-biotinylated mAb 7-B6-1 (Mabtech, Stockholm, Sweden) in PBS was added and the plates were incubated for an additional 3 h at room temperature. The plates were again washed and 50 µl/well of streptavidin alkaline phosphatase (Mabtech, Stockholm, Sweden) diluted 1∶1,000 in PBS was added for 1 h at room temperature. After washing again, the spots were visualized by adding 100 µl/well of substrate, 5-bromo-4-chloro-3-indolylphosphate and nitro blue tetrazolium (BIORAD) for 5 min. The number of specific IFN-γ secreting T-cells was counted using an automated ELISpot reader (Carl Zeiss, Germany). Calculation was done by subtracting the average negative control value and expressed as spot forming cells (SFC) per 10^6^ input cells. A response was considered positive if it was greater than (a) 3× the mean background, (b) the mean background +3 standard deviations or (c) 50 SFC per 10^6^ input cells, whichever was higher. CD8+ T-cell dependence of the optimal YI9 response was confirmed by CD8 depletion.

### Cytotoxicity assays

5×10^5^ autologous BLCL were pre-incubated with peptides for 1 h and later labeled with 80 µCi of Na_2_(^51^CrO_4_) (New England Nuclear, North Billeicas, Mass.) for 1 h. After incubation, the cells were washed three times with RPMI 1640 medium. The ratios of effector to target (E∶T) cells were 30∶1 and 15∶1. Cytolytic activity was determined in a standard 4 h ^51^Cr-release assay using U-bottomed microtiter plates containing 5×10^3^ targets per well. Assays were done in triplicate. For maximum (max) release, 5% triton-X 100 (TX 100; Sigma) was added to the target cells. ^51^Cr release from wells with culture media R10 only was used to calculate the spontaneous release (min). One hundred microliters of supernatant were counted on a gamma counter (Wallac, Gaithersburg, MD). Results were reported in counts per minute (CPM). Percent lysis was determined by the following formula: % specific lysis = [(mean CPM Test−mean CPM spontaneous)/(mean CPM Test−mean CPM Max)]×100. Spontaneous release values were less than 30% of maximal release for all reported assays. For the peptide titration assays target cells were pulsed with serial 10-fold dilution, starting from a peptide concentration of 100 µg/ml down to 1 pg/ml (−4 to −12 log molar).

### HLA-restriction and fine mapping

HLA restriction was performed using autologous or partially HLA-matched target cells pulsed with peptide in a ^51^Cr-release assay. Cells without any added peptide served as negative controls. Fine mapping of the optimal epitope was performed using truncations and extensions of the predicted optimal epitope using both ELISpot and ^51^Cr-release assay. ^51^Cr-release assays were performed at effector to target cell ratios of 30∶1 and 15∶1.

### Sequencing

Autologous virus was sequenced from plasma RNA using population sequencing as described previously [46]. HIV-RNA was extracted from plasma using QIAmp RNA Viral Mini kit (QIAGEN, Germany) and nested PCR was performed using primers specific for clades B and CRF01_AE. For the first round of PCR amplification of Gag a combination of three primer pairs were used: 276F-GAGGTGCACACAGCAAGAGGCG with 1972-R CCCCCTATCATTTTTGGTT-TCC, 623F-AAATCTCTAGCAGTGGCGCCCGAACAG with 2827R-TAACCCT-GCGGGATGTGGTATTCC and 737F-GCGRCTGGTGAGTACGCC with 2110R-RGGAAGGCCAGATYTTCC. Second round nested amplification was conducted using the primer pair 294F-GGCGAGAGCGGCGACTGGTGAG with 1920R-CTGTAT-CATCTGCTCCTGTATC. First round PCR cycling conditions were as follows: 95°C for 15 minutes followed by 35 cycles of 30 s at 94°C, 30 s at 54°C, 2.25 min at 72°C and a final extension of 68°C for 20 min. Nested PCR reactions were 95°C for 2 minutes followed by 35 cycles of 30 s at 94°C, 30 s at 58°C, 1.75 min at 72°C and a final extension of 68°C for 20 min. PCR fragments were then sequenced bi-directionally on an ABI 3130 automated sequencer using previously published primers [47]. Sequencher (Gene Codes Corp., Ann Arbor, MI) and MacVector 4.1 (Oxford Molecular) were used to edit and align the sequences. Sequences are submitted under Genbank numbers HM627654-HM627753.
